# Domestic and intimate partner violence among pregnant women in a low resource setting in South Africa: a facility-based, mixed methods study

**DOI:** 10.1186/s12905-018-0612-2

**Published:** 2018-07-04

**Authors:** Sally Field, Michael Onah, Thandi van Heyningen, Simone Honikman

**Affiliations:** 1 Perinatal Mental Health Project, Alan J. Flisher Centre for Public Mental Health, Department of Psychiatry and Mental Health, University of Cape Town, Building B, 46 Sawkins Road, Rondebosch, Cape Town, 7700 South Africa; 2 School of Public Health and Health Systems, University of Waterloo, Waterloo, Canada

**Keywords:** Intimate partner violence, Domestic violence, Abuse, Low-income setting, South Africa, Mixed methods, Mental disorders, Pregnancy, Food insecurity, Antenatal

## Abstract

**Background:**

Rates of violence against women are reported to be highest in Africa compared to other continents. We aimed to determine associations between mental illness, demographic, psychosocial and economic factors with experience of intimate partner violence (IPV) among pregnant women in a low resource setting in Cape Town and to explore the contextual elements pertaining to domestic violence.

**Methods:**

We recruited adult women attending antenatal services at a primary-level maternity facility. Demographic, socioeconomic and psychosocial data were collected by questionnaire. The Expanded Mini- International Neuropsychiatric Interview (MINI) Version 5.0.0 was used to assess mental health status and the Revised Conflict Tactic Scale (CTS2) used to assess IPV in the six months prior to the study. Non-parametric tests, Wilcoxon sum of rank test, Fisher Exact and two sample T test and multicollinearity tests were performed. Descriptive, bivariate and logistic regression analyses were conducted to identify associations between the outcome of interest and key predictors. A probability value of *p* ≤ 0.05 was selected. From counselling case notes, a thematic content analysis was conducted to describe contextual factors pertaining to forms of domestic violence (DV).

**Results:**

The prevalence of IPV was 15% of a sample of 376 women. Women who were food insecure, unemployed, in stable but unmarried relationships, had experienced any form of past abuse and were not pleased about the current pregnancy were more likely to experience IPV. MINI-defined mental health problems and a history of mental illness were significantly associated with IPV. Qualitative analysis of 95 counselling case notes revealed that DV within the household was not limited to intimate partners and, DV in this context was often perceived as ‘normal’ behaviour by the participants.

**Conclusions:**

This study contributes towards a greater understanding of the risk profile for IPV amongst pregnant women in low-income settings. Adversity, including food insecurity and mental ill-health are closely associated with IPV during the antenatal period. Advocates against violence against pregnant women are advised to consider that violence in the home may be perpetrated by non-intimate partners and may by enabled by a pervasive belief in the acceptability of the violence.

## Background

Rates of violence against women are reported to be highest in Africa compared to other continents [1]. In South Africa, the national mortality rate attributed to intimate partner violence (IPV) was found to be double that of the United States [2]. A World Health Organisation multi-country study on IPV found that between 15 and 71% of women reported lifetime physical or sexual violence by a partner [3]. This is an important public health concern.

Domestic violence (DV) is defined as any physical, sexual, psychological or economic abuse that takes place between people who are sharing, or have recently shared a residence [4]. While this usually takes place between intimate partners [5], in the context of a low-resource setting, where extended family members reside within the same household, DV is not limited to intimate partners. Globally, rates of DV seem to be higher in rural than urban areas, with most cases not being reported to police or healthcare providers. Thus, data reported in epidemiologic studies are likely to underestimate the prevalence [6].

A national study conducted in the USA, with data from over 34,000 participants, showed that DV is associated with physical illness including injury, chronic pain, asthma and sexually transmitted infections [7]. In an economic evaluation of the cost of IPV to the Australian health system, it accounted for 8% of the overall burden of disease for women of child-bearing age, contributing more than either raised blood-pressure, obesity or tobacco use. The association with poor mental health was one of the greatest contributors to this burden [8]. Globally, high levels of symptoms of perinatal depression, anxiety, and Post-traumatic Stress Disorder (PTSD) are significantly associated with having experienced DV [8–10]. Greater severity of traumatic experience and sexual violence are each associated with greater levels of depressive symptoms [11].

A Nigerian study reported that co-wives and step-sons perpetrate 24 and 17% of DV respectively [12]. In Bangledesh and Uganda, acid throwing is a commonly reported method of violence against women, usually perpetrated by family members other than partners [13]. In a South African study, Hoque et al. (2009) found that 12% of DV that occurred during pregnancy, was perpetrated by a family member other than a partner [14]. An earlier South African study indicated that 24% of DV during pregnancy was perpetrated by an in-law [15].

Violence during pregnancy has negative implications for both the mother and the child. In its most severe form, violence against pregnant women has been reported as a contributing cause of maternal deaths [16]. Violence during pregnancy has been associated with inadequate uptake of antenatal care, with abused women being more likely to delay seeking pregnancy care and to attend fewer antenatal visits [17]. Detrimental perinatal physical health outcomes for both mother and child have been reported. These include: low birth weight, foetal death by placental abruption, antepartum haemorrhage, foetal fracture, rupture of the uterus and premature labour [11] A strong association between suicidal ideation and IPV in pregnant women has been reported in low-income settings [18].

Many of the risk factors for violence during pregnancy have also been identified in studies of IPV and DV against women in general. However, global literature indicates that both unintended and unwanted pregnancies are each associated with experiencing violence during pregnancy [19]. A Canadian study showed that women with unintended pregnancies are three times more likely to experience IPV than those with intended pregnancies [20]. From global literature, other risk factors pertaining specifically to the antenatal period include: low socio-economic status, being young or adolescent, being unmarried, becoming separated or divorced during pregnancy, belonging to an ethnic minority, alcohol misuse by either the woman or her partner and low educational status [19, 21]. Further factors associated with IPV in Southern Africa, have been identified as: having a younger male partner, problem drinking by the partner, partner control of woman’s reproductive health, and risky sexual practices [22]. In Africa, studies on Human Immunodeficiency Virus (HIV) diagnosis and status as risk factors for IPV are inconclusive [21]. However, sexual risk factors have been positively associated with experience of IPV. These included transactional sex, having more than five lifetime sexual partners and having multiple sexual partners [21]. Further, this review shows that one of most significant predictors of violence during pregnancy is a history of abuse (defined as experiencing abuse before the age of 15, abuse in the past 12 months and abuse at any point over one’s lifetime) [21]. However, the pattern of abuse may be influenced by pregnancy itself. A global review study indicated that between 13 and 71% of women who are abused during pregnancy reported an increase in the frequency and/or severity of violence during this time [19].

In South Africa, high levels of violence occur within a context of multiple contributing social dynamics. These include prominent patriarchal norms where masculinity is associated with defence of honour, harshness and risk taking [23]. Poverty and gender inequalities contribute to the structural determinants of violence [24].

The aim of this study is to determine the associations between mental illness, demographic, psychosocial and economic factors with experience of IPV among pregnant women in Hanover Park, Cape Town. We also aimed to explore the contextual factors associated with violence in the homes of those women who received mental health counselling.

## Methods

### Study design

This was a mixed methods study which used a facility-based cross sectional survey and retrospective client counselling case-notes to examine our research objectives.

Survey design: The facility-based survey was used to examine the associations between mental illness, demographic, psychosocial and economic factors with experience of IPV.

Qualitative case study: Counselling case-notes were used to investigate counselled clients’ experience of IPV and the context and impacts of DV, by intimate partners and other members of the household.

### Ethics

Ethical approval for this study was obtained from the University of Cape Town Human and Research and Ethics Committee (HREC REF: 131/2009) and from the Western Cape provincial Department of Health. After the study was explained to them verbally, participants provided written, informed consent to participate in the survey and follow-up counselling services, if required. Consent forms were available in English and local languages and were administered by a research assistant. The consent included giving permission for use of sociodemographic data and counselling notes for research purposes. Procedures for anonymity and data security were described to the participants who were reassured that should they choose to decline participation in the study, or withdraw at any stage, this would not jeopardise their access to maternity care or care from the counsellor.

### Setting

Hanover Park Midwife Obstetric Unit, Cape Town was the site for this cross-sectional study. The facility provides public obstetric services to Hanover Park and the immediate surrounding areas. Hanover Park is considered to be one of the most violent communities in Cape Town. Crime statistics from the local police precinct indicate that 1412 cases of violent crime (murder, attempted murder, assault with the intent to do grievous bodily harm, common assault and sexual crimes) were reported in 2015 [25]. Many of these crimes are gang related, and are linked to the prevalent social problems of poverty, alcohol and substance abuse [26]. The population density of the area is one of the highest in Cape Town, with a population of 35,000 in an area of approximately two square miles [27]. The unemployment rate is 41%, with 63% of households have incomes of less than R 3200 (US$ 320) per month [28]. The inhabitants of 70% of households do not own their own homes [27] .

### Survey

#### Recruitment of participants

A k = 3 sampling frame was used to recruit pregnant women arriving at the facility for their first antenatal visit. This sampling method involves the selection of participants from an ordered sampling frame [29]. Using this frame, we started by selecting a participant from a list of eligible women at random. The list was provided by the clinic of women who had booked for antenatal services on any given day. Then, every third participant that presented herself for antenatal services was approached. The research assistant explained that the survey would be conducted in private and all information would be confidential, with no names or other identifying data attached to participants’ data. The recruitment strategy took account of the average number of women who presented daily for antenatal care, the amount of time that screening would take and included women who arrived at different times of the day. Consent was sought prior to women having their physical exam or their routine HIV test. Women included in the study were 18 years or older, willing to participate and able to understand the nature of the study, questions, and instructions given by the research assistant. Exclusion criteria included age (younger than 18), language (speaking a language other English, Afrikaans or isiXhosa), cognitive impairment, presenting with a false pregnancy, and having undergone HIV testing prior to being approached to participate in the study. Participants were recruited between November 2011 and August 2012.

#### Measurements

A socio-demographic questionnaire was used to collect information regarding asset ownership, age, language, education, marital status, obstetric information, whether the pregnancy was intended and wanted, as well as previous self-reported, mental health history. The section on asset ownerships asked yes / no questions for ownership of a list of 16 items that included: cell phone, fridge, vacuum cleaner, television, hi-fi, microwave, washing machine, DVD player, domestic worker services, flush toilet, built-in kitchen sink, electric hotplate or stove, car, shop at a supermarket, bank account and account on credit with a retail store. Household income data were collected and converted to United States Dollars [28].

The Revised Conflict Tactic Scale (CTS2) was used to assess IPV among the study population. The CTS2 is a condensed form of the original Conflict Tactics Scale and has been used in low-income countries to screen for IPV amongst women [30]. This tool has good reliability and validity. High alpha coefficient reliability was shown across 33 diverse sites when the tool was tested in 17 countries, including in low and middle-income settings [31]. The scale is used to measure physical assault, physical injury, psychological aggression, sexual coercion and negotiation [31] and has been used in cross-cultural studies in South Africa [9]. The seven questions have yes or no answer options, indicating the presence or absence of different forms of IPV over the six months preceding the study. A ‘yes’ answer to any of the questions indicates IPV. Positive responses to Questions 1–5 indicate forms of physical abuse, a positive response to question 6 indicates sexual abuse, and answering ‘yes’ to question 7 refers to emotional abuse.

The Multidimensional Scale of Perceived Social Support (MSPSS) was used to assess perceived social support from family, friends and a significant other [32]. This measure has been used previously in South African populations and has demonstrated good psychometric properties with good validity and reliability. [33, 34]. A Cronbach alpha of 0.86 was reported for the scale in a South African setting, and confirmed with factor analysis with reliability values within an acceptable range [34]. The Risk Factor Assessment (RFA) was used to assess for the presence of risk factors for psychological distress during pregnancy, and included a question on previous experience of any form of abuse,– termed ‘past abuse’ below. The RFA was developed by the Perinatal Mental Health Project (PMHP) [35], based on their local clinical practice and the common risk factors for perinatal psychological distress and depression identified in scientific literature [36, 37]. It consists of a checklist of 11 yes / no questions, each item assessing the absence or presence of a risk factor. In order to counter response bias, questions are phrased so that ‘yes’ indicates the presence of a risk for some items, but the absence of a risk for other items. It is used in clinical practise by PMHP counsellors [38, 39].

Food security was assessed using the revised Household Food Security Survey Module (HFSSM) which was developed to assess household hunger and food security in surveys [40]. This tool was used to collect information for the period of time six months prior to the survey [41]. The tool was designed to assist USAID implementation partners in collecting data regarding food insecurity in order to reduce hunger, malnutrition and food insecurity in developing countries [41]. Currently there is no standardised measure or tool to assess food insecurity in South Africa. The user notes of the short 6-item US household food security module suggests cut-points [37]. These were adopted and used as follows: a score of 0–1 indicates high or marginal food security; a score of 2–4 indicates low food security; a score of 5–6 indicates very low food security or food insufficiency.

The Mini-International Neuropsychiatric Interview (MINI Plus Version 5.0.0) was used as a diagnostic interview to assess common mental disorders (Major Depressive Episode (MDE), and anxiety disorders), suicidal ideation or behaviour and alcohol abuse and drug use [42]. It is an accepted diagnostic tool, and has been validated for use in South Africa [43, 44]. When the MINI was tested for inter-rater and test-retest reliability against the CIDI, Kappa coefficient, sensitivity and specificity were good or very good for all diagnoses [45].

All tools were translated and back translated from English into local languages, Afrikaans and isiXhosa. Interviews and counselling were conducted in any of these languages depending on the participant’s preference.

#### Data collection and management

A research assistant administered the socio-demographic questionnaires. Thereafter, a registered mental health counsellor, with a four-year tertiary qualification in counselling, administered the Expanded Mini- International Neuropsychiatric Interview (MINI) Version 5.0.0 diagnostic tool. Those women who were identified at screening (EPDS score of 13 or more or with three or more risk factors on the RFA), as having a mental health problem were offered free-of-charge, on-site counselling with a counsellor, at a time convenient for the women. Women who were offered counselling were not obliged to attend counselling sessions. However, referring staff were trained to facilitate uptake of counselling by assisting with scheduling to overcome possible logistical barriers. All women reporting domestic violence were provided with information regarding domestic violence and relevant available resources, irrespective of their uptake of counselling.

Women with features of psychosis or suicidal ideation, were immediately referred to the emergency unit at the community health centre, on the same premises. The research study counsellor offered supplementary counselling to these women, after their immediate crisis was managed.

Participants were provided with refreshments during the interview, but were not financially compensated for their time or reimbursed for travel costs.

Once each form had been completed and prior to data capture, survey questionnaires were manually logged by the research assistant and entered into an Access database. She used research identification numbers that had been assigned by the research co-ordinator. These ten-digit numbers represented the data collection date with the last two digits indicating consecutive number of participants completing the questionnaires on that day. Personal identifiers were removed to ensure confidentiality. The study co-ordinator held weekly supervisory sessions with the research assistant to cross-check the logs and the electronic database. These records were verified by the PMHP Project co-ordinator on a monthly basis. The study co-ordinator cleaned the data prior to data analysis. Access to the study data was limited to the researchers.

#### Data analysis

Quantitative data were analysed using Stata v13.1. [46]. Descriptive statistics were used to explore the characteristics of the study population. Households were grouped into quartiles based on their socioeconomic status as ascertained by an asset index. This method was devised to compliment the household income data because of the weaknesses inherent in collecting household income data (recall bias, under-reporting, accuracy, and lack of sensitivity to non-cash income) [47]. While constructing the asset index, information on ownership of electronic equipment, transport, sources of energy, and bank accounts were combined. This information was gathered from the socio-demographic questionnaire. In the principal component analysis, the first component factor was used to represent the asset index and based on this analysis, the study population was categorised into 4 quartiles (i.e., least poor, poor, very poor and poorest). Statistically, the first component factor is defined as the weighted sum of the different assets used to measures household wealth, in order for that component to explain as much as possible of the variance observed in asset ownership between households.

Internal consistency and scale reliability within assessment tools were assessed using the Cronbach’s α Statistics [48]. Statistically significant associations were examined using non-parametric tests, the Wilcoxon sum of rank test, the Fisher exact test and the two sample t-test where appropriate. Bivariate analyses were performed to examine associations between independent and predictor variables. Logistic regression was also employed to examine the associations between independent and predictor variables and results are presented as unadjusted odds ratios (OR). To adjust for potential effects of confounding variables, we constructed an adjusted logistic regression model. Variables included in this model were those that had significant odds ratios in the unadjusted models as well as variables that has been identified as risk factors from the scientific literature and PMHP practice [35]. We also assessed for correlations and multicollinearity among independent predictor variables within the regression model [49]. Independent variables that were considered to have endogeneity characteristics with the dependent variable were excluded from the model. Results are presented as adjusted odds ratios (aOR).

### Qualitative study

#### Selection of case notes

Counselling case notes were retrospectively examined. The eligible case notes were from women who had been recruited into the survey, referred for mental health support and had received and completed counselling for mental health problems. The research study’s mental health counsellor, a woman, provided face-to-face, therapeutic counselling support to referred participants during this time period. Referral for counselling was not contingent on completion of the survey questionnaire, but rather on being assessed with a mental health problem on the EPDS with a score of 13 or more, or of having three or more risk factors on the RFA. The counselling sessions took place in a private room at the clinic. Each counselling session lasted approximately 50 min. There was no limit on the number of counselling sessions per client. The initial assessment session included open-ended exploration of current and prior experiences of violence which the counsellor then documented in her case notes. The assessment form provided a check box for experience of domestic violence. When ‘Yes’ was ticked for domestic violence on the assessment form, the case notes were selected for qualitative analysis.

#### Measurements

Detailed case notes, written by the mental health counsellor during her counselling sessions with clients were used as a proxy representation of women’s experience and accounts of domestic violence.

#### Data collection and management

Qualitative data were collected from detailed counselling case notes. Confidentiality was maintained as the counselling notes were securely stored with access restricted to the counsellor and the researchers. In order to further protect client anonymity, research identification numbers, were assigned by the researchers prior to charting the case notes. Personal identifiers were removed.

Informed by a review of exiting literature on types of abuse and also from PMHP’s experience of providing mental health services for 15 years, two researchers created a priori categories for the framework matrix. These categories were: emotional / verbal abuse, physical abuse, sexual abuse and substance use. The researchers then reviewed the notes that indicated experience of DV, looking for key words indicating type of abuse (emotional / verbal, physical, sexual) and substance use. Data were charted into an Excel document under key word categories by a data capturer who transcribed the notes verbatim. The document was then verified against the notes by a researcher to ensure the accuracy of the charting and that all relevant material had been captured.

#### Data analysis

A thematic content analysis, using the framework method was conducted for all the extant counselling case notes, focussing on domestic violence [50]. This method has been used increasingly for qualitative health research and is appropriate for use with existing text-based sources, such as case notes [51]. Two researchers reviewed and coded the notes independently. The major themes and sub-theme that emerged from the texts were arrived at by each researcher independently and consensus reached.

## Results

### Facility-based survey results

During the data collection period, 2228 women booked for antenatal care at the facility. 562 eligible women were approached to participate, of which 55 (9%) declined. Reasons cited were predominantly logistical: they did not have the time or child care arrangements to engage with the interviewer. A further 140 women did not complete the full survey, due to time-constraints or feeling tired, and these data were excluded from the survey database. In total, three hundred and seventy-six (376) women (67% of those approached) contributed survey data.

While 26% were experiencing their first pregnancy, 30% were in their second, and 45% had had multiple pregnancies. Only 6% of the sample were single, with 4% in casual relationships and the majority (51%) in stable but unmarried relationships. The remaining 39% were married. Sixty percent of the sample had attained an education level of grade 10 or more. Over half of the sample (58%) were unemployed at the time of the study. Based on their asset index, 2% belonged to the poorest socio-economic status index while 25% belonged to the least poor category. Indicated by the RFA questionnaire, approximately a quarter of the sample (24%) reported experiencing some form of abuse (emotional, physical or sexual) in the past.

From the CTS2 tool, the prevalence of IPV was 15% (*n* = 58) of the 376 sample population. Of those reporting IPV, 81% (47/58) of women reported emotional and verbal abuse (endorsement of question 7), 76% (44/58) reported physical abuse (endorsements of questions 1–5) and 26% (15/58) reported sexual abuse (endorsement of question 6). Furthermore, 46% of individuals that screened positive for IPV had experienced multiple forms of abuse. Forty-nine percent of the women that experienced this violence were between 18 and 24 years of age. Over half (62%) had education levels of Grade 10 and lower. The majority were in the second trime*s*ter of their pregnancy. Of those that experienced IPV, 58% were not currently employed. Food insecurity was noted for 62% of these women. Out of the total number of women currently abused by a partner, 66% were in a stable relationship but not married, 50% had experienced a form of past abuse, and 40% were not pleased with their current pregnancy. Of the sample experiencing IPV, 40% were diagnosed with MDE, 36% with an anxiety disorder and 31% were assessed with suicidal thoughts or behaviours. Twenty-nine percent of these women used alcohol and other drugs and 24% had a self-reported history of mental health problems.

Results of the Cronbach’s α tests show that the CTS2 tool (Cronbach’s α0.85), the MSPSS tool (Cronbach’s α 0.89), and the HFSSM tool (Cronbach’s α 0.83) exhibited good internal consistency and reliability when used on the study sample. Results of the multicollinearity test indicated that the variance inflation factors among the predictor variables were below 10 points and the regression coefficients exhibited stability with a condition number below 10 points. This shows that there is no multicollinearity inherent in the model.

Bivariate analyses generated significant variables that were incorporated into the multivariable model. See Table 1.

**Table 1:** Characteristics for survey participants and bivariate associations between IPV and demographic and psychosocial factors

Participant characteristics	Total (*n* = 376) n (%)	Intimate Partner Violence	
		Positive (*n* = 58) n (%)	Negative (*n* = 318) n (%)
Age: 18–24 years	146 (39)	28 (49)	118 (37)
25 29 years	114 (30)	21 (36)	93 (29)
> 29 years	116 (31)	9 (15)	107 (34)*
Parity: Nulliparous	122 (32)	23 (40)	99 (31)
Primiparous	128 (34)	19 (33)	109 (34)
Secundiparous	83 (22)	9 (15)	74 (23)
Multiparous	43 (11)	7 (12)	36 (12)
Gravida: Primigravida	96 (26)	18 (32)	78 (24)
Secundigravida	114 (30)	20 (34)	94 (30)
Multigravida	166 (45)	20 (34)	146 (46)
Gestation: 1st trimester	96(32)	14 (32)	82 (32)
2nd trimester	175 (58)	27 (61)	148 (58)
3rd trimester	29 (10)	3 (7)	26 (10)
Education level (≥Grade 10)	225 (60)	36 (62)	189 (59)
Working currently	159 (42)	16 (28)	143 (45)*
Socio Economic Status: Least poor	94 (25)	16 (28)	78 (25)
Poor	94 (25)	15 (26)	79 (25)
Very poor	96 (26)	11 (19)	85 (27)
Poorest	91 (24)	16 (28)	75 (24)
Food insecure	158 (42)	36 (62)	122 (38)**
Relationship type: Married	146 (39)	14 (24)	132 (42)
Stable partner	192 (51)	38 (66)	154 (49)
Casual partner	16 (4)	2 (3)	14 (4)
Single	22 (6)	4 (7)	16 (5)
Perceived support from family	23 (5)	21.86 (SD 5.23)^a^	22.66 (SD 5.04)^a^
Perceived support from friends	20 (7)	18.34 (SD 6.80)^a^	20.30 (SD 6.70)^a^
Perceived support from “special person”	24 (4)	22.62^a^ (SD 4.87)^a^	24.33^a^ (SD 3.68)^a^
Past abuse^b^	89(24)	29 (50)	60 (19)**
Not pleased with pregnancy	81 (22)	23 (40)	58 (18)**
Major Depressive Episode (MDE)	81 (22)	23 (40)	58 (18)**
Any anxiety disorder	86 (23)	21 (36)	65 (20)*
Suicidal Ideation or Behaviour (SIB)	69 (18)	18 (31)	51 (16)*
Alcohol and other drug use (AoD)	65 (17)	17 (29)	48 (15)*
History of mental health problems	57 (15)	14 (24)	43 (14)*

*significant at p ≤ 0.05, **significant at p ≤ 0.01

^a^ *mean score*

^b^*From item on RFA “I have experienced some kind of abuse in the past* e.g. *physical, emotional, sexual, rape”*

Demographic and economic factors associated with IPV were also explored. Significant findings showed that women older than 29 were less likely to report an experience of IPV than younger women (aOR 0.25, 95% CI 0.09–0.65). Pregnant women who were food insecure were more likely to report an experience of IPV than those that were food secure (aOR 1.96, 95% CI 1.01–3.76). Pregnant women in a stable relationship but not married were twice as likely to report experiencing IPV than those who were married (aOR 2.48, 95% CI 1.17–5.27). As perceptions of social support from a “special person” increased, pregnant women were less likely to report an experience of IPV relative to those who experienced a lower perception of social support (aOR 0.91, 95% CI 0.82–0.99). Pregnant women that reported experience of any form of abuse in the past were four times more likely to report an experience of current IPV relative to women with no history of abuse (aOR 4.81, 95% CI 2.28–10.12). Women who were not pleased with their pregnancy were also twice as likely to report an experience of IPV compared to women who were pleased with their pregnancy (aOR 2.54, 95% CI 1.28–2.04). See Table 2.

**Table 2:** Unadjusted an adjusted multivariable associations between IPV and risk factors

	OR (95% CI)	aOR (95% CI)
Parity: Nulliparous	1	
Primiparous	0.75(0.38–1.46)	
Secundiparous	0.52(0.22–1.59)	
Multiparous	0.83 (0.33–2.11)	
Gravida: Primigravida	1	
Secundigravida	0.92(0.45–1.86)	
Multigravida	0.59(0.29–1.18)	
Gestation: 1st trimester	1	
2nd trimester	1.08(0.53–2.15)	
3rd trimester	0.67(0.18–2.53)	
Age: 18–24 years	1	1
25 29 years	0.95 (0.50–1.78)	0.99 (0.48–2.05)
> 29 years	0.35 (0.16–0.78)*	0.25 (0.09–0.65)*
Education level (≥Grade 10)	1.11 (0.62–1.98)	
Working currently	0.46 (0.25–0.86)*	0.26 (0.15–1.66)
Socio Economic Status: Least poor	1	1
Poor	0.92 (0.42–2.00)	1.11 (0.46–2.68)
Very poor	0.63 (0.27–1.44)	0.81 (0.32–2.06)
Poorest	1.04 (0.48–2.22)	1.05 (0.44–2.49)
Food insecure	2.62 (1.47–4.67)**	1.96 (1.01–3.76)*
Relationship type: Married	1	1
Stable partner	2.23 (1.20–4.48)*	2.48 (1.17–5.27)*
Casual partner	1.34 (0.27–6.54)	0.99 (0.17–5.76)
Single	2.35 (0.69–8.03)	1.72 (0.42–7.08)
Perceived support from family	0.97 (0.92–1.02)	1.06 (0.98–1.45)
Perceived support from friends	0.96 (0.92–0.99)*	1.00 (0.95–1.05)
Perceived support from “special person”	0.89 (0.86–0.97)**	0.91 (0.82–0.99)*
Past abuse^a^	4.30 (2.39–7.72)**	4.81 (2.28–10.12)**
Not pleased with pregnancy	2.94 (1.61–5.35)**	2.54 (1.28–2.04)**
MINI assessed mental health problem	2.7 (1.51–4.80)**	1.33 (1.25–2.69)*
History of mental health problems	2.03 (1.02–4.02)*	1.93 (1.20–2.17)*

*significant at p ≤ 0.05, **significant at p ≤ 0.01

^a^*From item on RFA “I have experienced some kind of abuse in the past* e.g. *physical, emotional, sexual, rape”*

Results also indicated that IPV was associated with MINI-defined mental health problems. These included MDE, any anxiety disorder, suicidal ideation or behaviour (SIB) and alcohol and substance use disorders (AOD). We grouped these assessed mental health problems into one variable because of the inherent correlation between the different types of assessed conditions and their impact on the adjusted logistic regression. Women with an assessed mental health problem were more likely to have reported experiencing IPV relative to those with no assessed mental health problem (aOR 1.33, 95% CI 1.25–2.69). Women who had a self-reported history of mental health problems were also more likely to report an experience of IPV than those with no history of mental health problems (aOR 1.93, 95% CI 1.20–2.17). See Table 2.

### Qualitative results

From the case notes of the qualitative study sample (*n* = 95), 31 women were noted to have been experiencing domestic violence in the current pregnancy, 55% (17/31) by someone in the household who was not an intimate partner. Perpetrators included fathers, stepfathers, uncles, brothers, grandmothers and brothers in-law. Six of these 17 women were simultaneously in a current relationship with an abusive partner. Sexual abuse was reported by 23% (7/31), 48% (15/31) reported verbal / emotional abuse and 74% (23/31) reported physical abuse. Further, 38% (12/31) reported a history of abuse.

Although the focus of the study using the quantitative data was to explore the predictors and associated risk factors for domestic violence, the client case notes provided additional information. The client notes raised themes that were not apparent from the quantitative data. This is summarised in Table 3. The main themes that emerged were: alcohol and substance abuse by members of the family were a contributing factor to violence; past abuse affected current behaviours and violence was seen as “normal behaviour” for many of the participants. The case note examples in Table 3 below add detail to the themes. A cross cutting sub-theme to emerge was the wide diversity and forms of abuse, many of them physical and escalating. They included: not providing food, social isolation, swearing, shouting, smacking, beating with fists, hitting with objects, stabbing, and forced sex.

**Table 3:** Case note themes and examples

Theme	n respondents (n = 31)	Case note examples
Alcohol and substance abuse by members of the household as a contributing factor to violence	19	A: “Her stepfather started drinking excessively and would beat the [participant’s] mother in front of the children. Current boyfriend drinks excessively.”
		B: “Husband is drinking excessively. Stays away for long periods of time without telling her of his whereabouts. He borrows money from other people to obtain alcohol. He came home drunk after being away the whole day. She was so angry she smacked him. This started a fight.”
Past abuse affecting current behaviours	12	C: “Witnessed [participant’s] mother’s ex-husband beating her mother. Gets flashbacks. She pictures the husband beating her mother and becomes extremely angry.”
		D: “Abused as a child, raped, sodomized/abused by ex-husband.”
Violence is “normal behaviour”	16	E: “He hit her against her head and hit her with a fist against her stomach. This is how they normally handle conflict. It doesn’t seem strange/abnormal that they are so violent with each other.”
		F: “She and her husband often get into physical fights with each other. An argument inevitably leads to fighting.”

## Discussion

In our sample of pregnant women, 15% were experiencing IPV and this was associated with food insecurity, unemployment, unmarried, but stable relationship status, past experience of abuse and discontent with the current pregnancy. Current mental health diagnosis and a self-reported history of mental illness were also significantly associated with IPV. Domestic violence within the household was not limited to intimate partners and, domestic violence in this context was often perceived as ‘normal’ behaviour by the participants.

The prevalence of IPV in this study was 15% using the CTS2 tool, which is considerably lower than the finding from a survey of pregnant women attending a public sector South African antenatal service. That study reported 38% had experienced abuse from a partner at some point in their lives, with 35% reporting domestic violence during the current pregnancy [15]. It is possible that women who experience mental health problems or domestic violence may be more likely to decline participation in a study of this nature. However, this does not explain the discrepancy in the findings of the two studies [15]. The authors of the Mbokota study, both health professionals, indicated that they used directed interviews to investigate the presence of domestic abuse. It is possible that this method was perceived by participants as a more confidential space than a survey questionnaire administered by a research assistant, and thus increased disclosure. Other literature supports increased detection via face-to-face screening [52] and further increased identification of DV when this was done by a health professional [53]. The prevalence in our study is the same as the overall prevalence yielded by a meta-analysis review on IPV during pregnancy in Africa [21]. However, while the case study sample size was very small, the prevalence of DV was higher than that reported by the survey sample: 32% as opposed to 15%. Several factors could contribute to this difference. The case study notes were from women who had received counselling for mental health problems and would therefore have been a high risk group. The association between mental illness and IPV has been reported in South Africa [54–56], as well as in global studies [8–10]. Further, women may have been more open to disclosure of DV during counselling than in a survey. Other studies have demonstrated that asking open-ended questions about DV are more likely to elicit disclosure [57, 58]. In addition, women from our case note sample reported violence within the household, of which, 55% was perpetrated by a non-intimate partner. Another South African study reported high levels of domestic violence during pregnancy, with 24% of the abuse being perpetrated by the mother-in-law of the pregnant woman [15]. This underscores the need to screen for domestic violence as perpetrated by other members of the household, and not only by intimate partners.

We found that women who were unemployed were more likely to have reported experiencing violence from their partners. While the settings are not the same, these findings contrast with a study conducted in Ugandan that found no difference in the experience of abuse between women who were unemployed and those who were employed in either the formal or informal sectors [50]. Further, we found that food insecurity was associated with IPV. Shamu et al. assert that, in Africa, the feminization of poverty means that many poor women rely on their partners for household maintenance and access to pregnancy care. Men who are perpetrators of physical violence exploit this economic vulnerability by abusing their partners [21]. This economic exploitation is a further abuse, in and of itself [4, 59].

We identified intimate relationship status and perceived support as significant factors associated with abuse. Women in stable but unmarried relationships were more likely to have reported experiencing intimate partner violence than those who were married. This is supported by findings from a United States (US) based study on low-income pregnant women [60]. Further, our findings concur with evidence from low and middle income countries that a lack of perceived support from a significant other, is significantly associated with IPV [19, 61]. Interventions targeting domestic violence may be enhanced by linking the survivors of violence to supportive networks and by assisting them in identifying and maximising any existing supportive relationships. We showed a significant association between those women who were not pleased about the current pregnancy and IPV. This is supported by findings in a review that consolidated findings from global literature [19] and has been found in a US study among pregnant women in a community setting [20]. We postulate that an unwanted pregnancy reflects dysfunction in interpersonal relationships and or compromised socio-economic status, both of which are themselves factors associated with domestic violence. Further qualitative investigation may elucidate the complexity of these interactions.

Current mental health problems were significantly associated with IPV in the bivariate analyses in this study. This association is supported by studies in both high and low-income settings [8–10, 18, 62] and by a systematic review and metanalysis [63] . Further, a South African, clinic-based study by Mbokota et al. indicated that 78% of the sample who experienced domestic violence during pregnancy experienced psychological problems [15], this is supported by our multivariable analyses, which indicated that women who reported experiencing violence were 2.4 times more likely to experience a mental health problem. However, the Mbokota study [15] failed to indicate how psychological problems were assessed and did not present diagnostic data for specific disorders. Chen et al. provide a systematic review on sexual abuse (only) for any women in any setting, with diagnostic psychiatric data. They found significant associations between a history of sexual abuse and diagnoses of depression, anxiety, eating and sleeping disorders and Post-traumatic Stress Disorder (PTSD). However, few of these studies were from low-income settings [64]. A community-based, South African study on pregnant women, linked depressive symptoms, obtained from screening data, with IPV and alcohol abuse [65]. The association of IPV with the use of alcohol and other drugs, either by the pregnant woman or by members of her household, is supported by other research from the US with urban, minority women [66, 67]. Our study adds to the literature from low-income settings by demonstrating associations with current mental health problems as assessed by diagnostic interview. Additionally, our finding that a history of mental health problems is significantly associated with IPV is widely supported by others’ evidence [8–10, 18, 62]. Although many of these studies, like ours, were not designed to demonstrate causal relationships, the evidence suggests that mental ill-health and violence against women exist in a vicious cycle, maintained by inequitable gender norms, low relationship power, poverty and the societal acceptability of violence [11]. This is supported by work conducted on social determinants of mental ill-health [68]. Machisa et al. described the structural pathways to IPV for a sample of South African women where binge drinking, depression, PTSD and lower relationship power mediated the relationship of prior childhood abuse and recent IPV [11]. Not excluding other factors, we hypothesise that the experiences typical of common mental disorders, i.e. low self-esteem, social withdrawal and a sense of helplessness [69, 70] may confer particular vulnerabilities to violence for pregnant women with these mental health problems. Mental ill-health, possibly through the same mood and cognitive features described above, also compromises women’s access to social and emotional resources [71, 72] which further maintains their vulnerability to victimisation. The co-existence of mental ill-health and violence against pregnant women has implications for the design of interventions for women who experience domestic violence.

In a review of studies on IPV from African countries, a history of experiencing abuse (abuse before the age of 15, abuse in the past 12 months and abuse in lifetime), is strongly associated with IPV during pregnancy [21]. While our qualitative sample size was low, and should not be considered as representative, the findings support this association, with examples of currently abused women reporting having been abused during childhood or by previous partners. For example, “Abused as a child, raped, sodomized/abused by ex-husband”.

The qualitative data from this study provide examples of how domestic violence was considered normative in this sample. This is reflected in the examples: “He hit her against her head and hit her with a fist against her stomach. This is how they normally handle conflict. It doesn’t seem strange/abnormal that they are so violent with each other.” and “She and her husband often get into physical fights with each other. An argument inevitably leads to fighting.” Several studies from low income settings have produced findings indicating the influence IPV has on social norms. Women who experienced family violence as a child are more likely to perpetrate violence [73], and find wife beating acceptable [74–77]. A WHO World Report on Violence and Health indicates that social norms and values play a powerful role in how violence is perceived, condoned, inhibited and responded to [78].

Benjamin (2014) draws on the concept of dysfunctional community syndrome, where violence takes many different forms and occurs increasingly over generations and at increased intensity, to describe the setting in Hanover Park [79]. Benjamin and Crawford-Browne (2011) refer to observed patterns of behaviours in clients from Hanover Park who attended counselling over a number of years, and were exposed to continuous trauma due to their violent environment. These clients had an inability to regulate emotion which was demonstrated by heightened or flattened affect in response, high levels of aggression, a reduced capacity for empathy and an inability to regulate impulsivity. There was also the tendency to minimise the impact of trauma [80]. The behaviours they describe seem to indicate how acceptable violence is in this environment, and is reflected in the examples provided in the qualitative descriptions of our study.

In the context of normalised violence, gender-based power disparities and poverty typical within the South African settings such as this [23], it is thus not surprising that domestic violence is perpetrated both by intimate partners as well as by other members of the household. Our finding that 55% of the case study sample had experienced domestic violence by a member of the household who was not an intimate partner concurs with other studies that have disaggregated the perpetrators of domestic violence in samples with pregnant women [12, 14, 15].

This demonstrates the need for intervention design to take account of motivations for violence and to deconstruct relationships of power and coercion. Further, our study reinforces the imperative to change societal norms regarding the acceptability of violence, and the need for preventive work and well as programmes that separately target men, women and children.

## Limitations

Our study experienced several limitations in design. Firstly, while the counsellor explored experience of domestic violence in her initial assessment, there was no routine structured format for this and therefore she may not have elicited information around frequency, progression, intensity or perception of violence. Further, the source of the qualitative data was the notes from one counsellor and thus represent her opinions and judgements. There is therefore the potential that some elements were unduly emphasized while others may have been minimized. This potential bias is mitigated by the fact that the counsellor had a four-year university qualification in counselling (Bachelors of Psychology), a formal registration with the national health professions council, had nearly a decade of experience working with clients experiencing high levels of violence, and received weekly individual clinical supervision by a clinical psychologist.

In addition, the definitions of violence across the quantitative and qualitative data differ, one taking into account intimate partner violence only, and the other using the broader definition of domestic violence. The cross-sectional nature of the quantitative data limits an investigation into the progression of violence before, during the pregnancy and after the birth, particularly as the RFA item pertaining to past abuse was unspecific. Further, it precludes interpretations concerning the directions of associations and causality. The study sample, was taken from one site and is relatively small and hence should not be generalised to other settings. All data collected were self-reported and we did not verify information such as asset ownership and prior self-reported mental health diagnosis. There is therefore room for recall bias and misreporting of information. Further, although uptake of antenatal services are high in South Africa (97%) [81], data were collected from service users of a clinic. This means that non-users of antenatal services, who might be the most vulnerable to domestic violence, would have not have been included in the sample.

## Conclusions and recommendations

The findings from this study have implications for service design and implementation. Detection of DV may be problematic, and should not be limited to IPV. Service designers need to be mindful of the possibility of under-detection. Further research may be needed to determine the best methods for this. Women who are more vulnerable to abuse may simultaneously have diminished access to resources due to poverty. Further, interventions should take into account the effects of complex household dynamics on relationships and the implications for safety for women and children.

When detecting domestic violence, we recommend simultaneous screening for mental disorders and the inclusion of mental health care into the design of interventions against domestic violence. In particular, interventions should take in to account the impact that mental distress may have in creating barriers to uptake of any services that are provided. For pregnant women, we would thus recommend social and mental health support for IPV and DV to be provided on-site and integrated into routine antenatal care offered by health centres. Additionally, broad-based interventions that assist with access to social grants or employment skills could assist with breaking the financial dependence on violent partners or other household members [82].

This study contributes towards a greater understanding of the risk profile for IPV amongst pregnant women in low income settings. Adversity including poverty and mental ill-health are closely associated with IPV during the antenatal period. We advocate for increased, holistic and contextually responsive interventions to assist those experiencing domestic violence from partners as well as other members of the household.

## Abbreviations

AOD: Alcohol and substance use disorders
aOR: Adjusted odds ratio
CTS2: Revised Conflict Tactics Scale
DV: Domestic Violence
HFSSM: Household Food Security Survey Module
HIV: Human Immunodeficiency Virus
HREC: Human Research Ethics Committee
IPV: Intimate partner violence
MDE: Major Depressive Episode
MINI: Mini International Neuropsychiatric Interview
MSPSS: Multidimensional Scale of Perceived Social Support
OR: Odds ratio
PTSD: Post-Traumatic Stress Disorder
RFA: Risk Factor Assessment
SIB: Suicidal ideation or behaviour
US: United States
